# A case of effective oral rehabilitation after mandibular resection

**DOI:** 10.1002/ccr3.2459

**Published:** 2019-09-27

**Authors:** Toshiyuki Kataoka, Yuichi Akagi, Chie Kagawa, Ryo Sasaki, Toshihiro Okamoto, Tomohiro Ando

**Affiliations:** ^1^ Department of Oral and Maxillofacial Surgery School of medicine Tokyo Women's Medical University Tokyo Japan

**Keywords:** grafted jaw bone, nonvascularized iliac bone, oral rehabilitation, osseointegrated implant, segmental mandibulectomy

## Abstract

Radical mandible resection causes significant functional and cosmetic impairment. Nonvascularized bone reconstruction and oral rehabilitation using fixed prosthesis with dental implants enabled recovery of appearance and mastication function.

## Graphical abstract

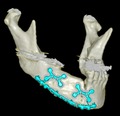

## 1 INTRODUCTION

Although removable dentures are provided for occlusal function recovery after mandible radical resection, patient satisfaction is poor. Therefore, we performed occlusal reconstruction for recurrent ameloblastoma with fixed prosthesis using osseointegrated implant on nonvascular iliac bone graft. The treatment results restored the patient's comfort and confidence.

Ameloblastoma is a typical odontogenic benign tumor that arises in the jaw bone. This tumor is known to be locally invasive and tends to recur. The need for radical resection of the mandible is determined according to the clinical behavior and radiopathological subtype of the tumor. Radical mandibulectomy results in asymmetry of facial features and difficulties with mastication, which worsen patients’ quality of life. Reconstruction with only soft tissue or only a mandibular titanium plate precludes occlusal reconstruction with a fixed prosthesis. The goal of mandibular reconstruction is to achieve aesthetic restoration by reproducing continuity of the mandible and to recover the functional cycle of swallowing and mastication. Reconstruction with fixed prosthetic osseointegrated implants is necessary for functional occlusion. We report a very effective oral rehabilitation case where occlusal reconstruction was performed using a dental implant on the nonvascularized iliac bone.

## 2 CASE REPORT

The patient was a 51‐year‐old woman who consulted our department in February 2013 about discomfort in the mandible. She had no medical history. Ameloblastoma was diagnosed during the first oral surgery in 2006, and she underwent fenestration procedures. The tumor recurred frequently, and she underwent repeated fenestration. She was dissatisfied with the outcomes of previous hospital's treatment policy and came to our department hoping for the complete resection of the tumor.

**Figure 1:**
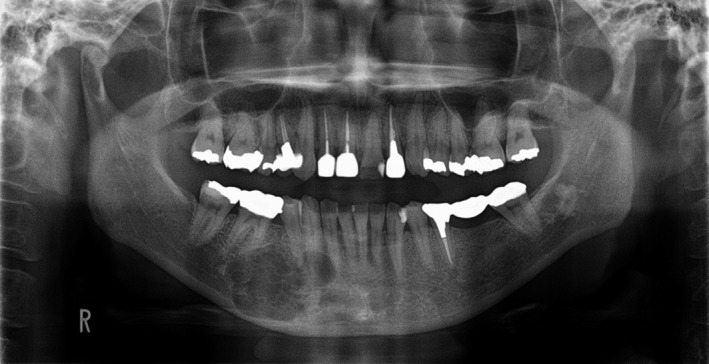
Orthopanoramic radiograph obtained during the patient's first visit. A multilocular radiolucency is visible in the right side of the mandible

**Figure 2:**
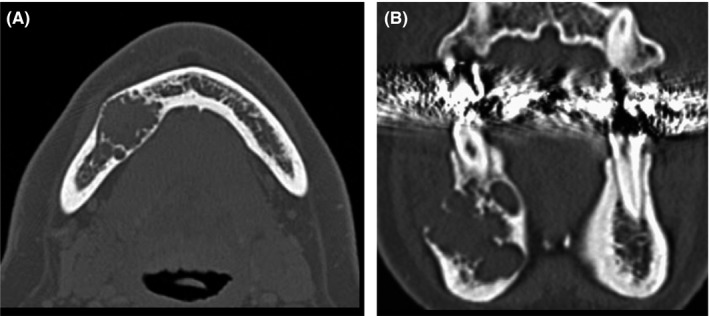
A, Preoperative computed tomographic image. Buccal and lingual expansion of the tumor is observed in the right side of the mandible. B, There is multifocal resorption in the right mandible

At the first visit, she described discomfort in the right side of the mandible and the perceptual abnormality of the mentum. Her face was symmetrical, but the mandibular right molar aspect bulged slightly; the swelling seemed like a parchment. On orthopanoramic radiographs (Figure 1), a multilocular radiolucent lesion was observed at the teeth position of 43 to 46 and extending to the lower edge of the mandible. Computed tomography (CT) images (Figure 2A, B) revealed that the right mandible was filled with tumor; multifocal resorption was evident, and the buccolingual aspect of the mandible was bulging. The inferior alveolar tube could not be distinguished from the lesions.

We diagnosed recurrence of ameloblastoma and proposed a treatment protocol of radical resection and immediate bony reconstruction. The graft donor site selected was a nonvascularized iliac bone. She consented to our surgical plan.

**Figure 3:**
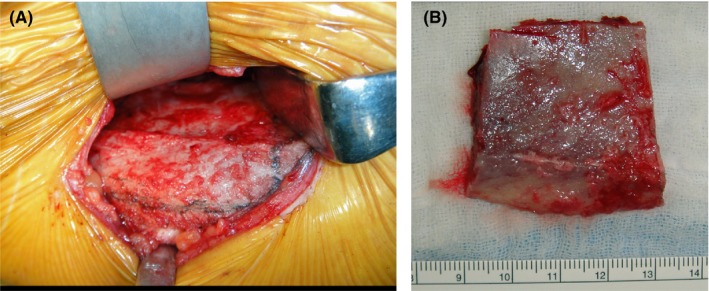
Intraoperative photographs. A, Design of iliac bone section to be harvested, outlined in marker. The inner plate was cutoff, and the iliac crest and the outer plate were preserved. B, Nonvascularized free iliac bone grafts (only inner plate) were harvested

**Figure 4:**
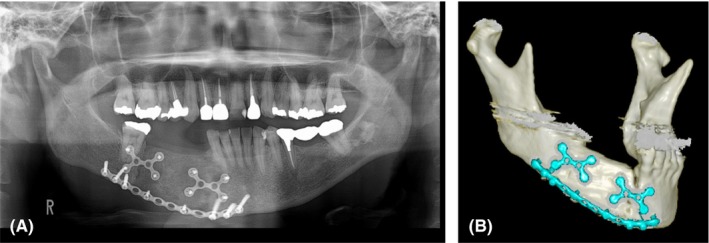
A, Postoperative orthopanoramic radiograph. B, The right mandible continuity defect was reconstructed with nonvascularized free iliac bone to correspond to the computed tomographic image

In March 2013, the patient, under general anesthesia, underwent right mandibular resection (teeth 43 to 46; length 35 mm) and reconstruction with nonvascularized free iliac bone. The mandibular defect classification^1^ was “L”: lateral defect without the condyle and not crossing the midline. Before resection of the mandible, a memory plate was installed to maintain the shape of the mandible. The memory plate was prepared prior to surgery by modifying the surgical model to mirror the healthy side of the mandible. A 4 × 4 cm^2^ section of the iliac bone was harvested at the inner plate (Figure 3). The iliac crest was placed at the lower rim of the mandible and affixed to a mini‐plate (Figure 4A, B).

**Figure 5:**
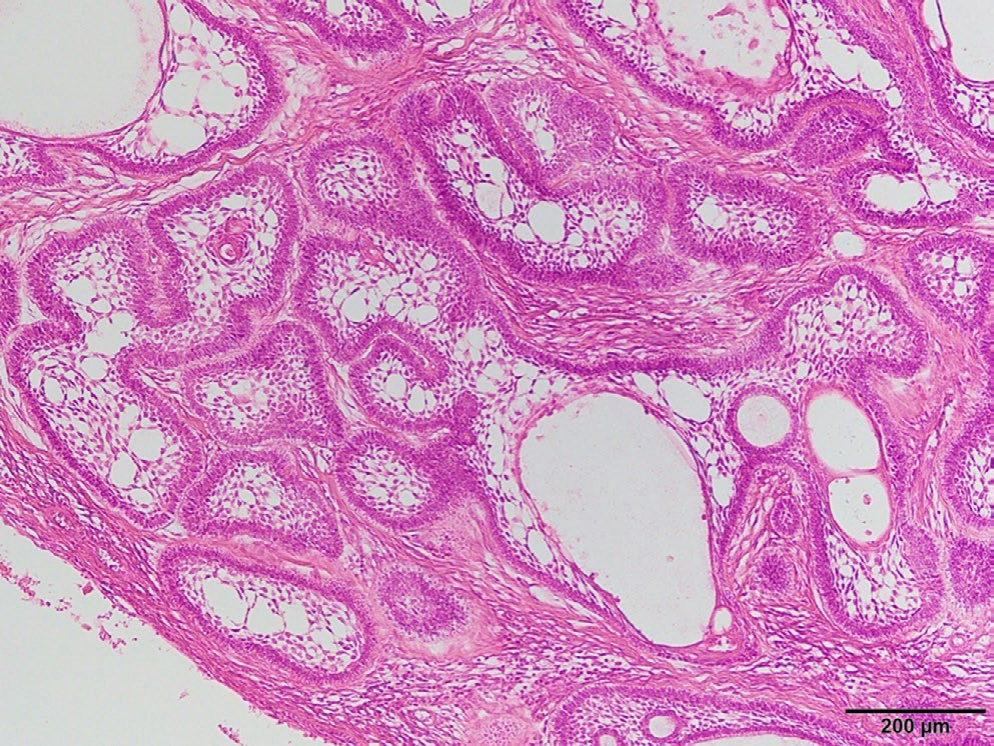
Histopathological appearance of the tumor. The tumor nests consisted of odontogenic epithelium with columnar peripheral cells. The inner cells resemble stellate reticulum

Histopathological study (Figure 5) revealed that the tumor nests comprised the peripheral cylinder layer and stellate reticulum, but had no mitotic figures and atypia. The final pathological diagnosis was ameloblastoma.

**Figure 6:**
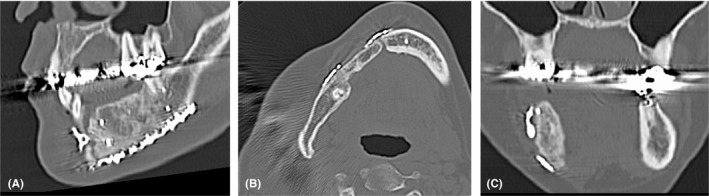
A, Sectional computed tomographic image obtained 1 y and 6 mo after surgery. It can be confirmed engraftment of bone graft. B, The grafted jaw bone is 30 mm high and 10 mm wide. It can satisfactorily accommodate a dental implant of root length. C, The osteosclerosis, similar to the cortical bone, can be identified around the graft bone

At follow‐up 1 year and 8 months after surgery, the tumor had not recurred. However, the patient was suffering from right‐sided difficulty in mastication; removal partial denture was not aesthetically preferable, and therefore, she wanted a fixed prosthesis. We decided to start occlusal construction of a fixed prosthesis with dental implants. The reconstructed jaw bone was 30 mm high and 10 mm wide, which was large enough to receive the dental implants (Figure 6A, B). Osteosclerotic findings like the cortical bone were observed around the grafted bone on CT images (Figure 6C). The occlusal relationship was examined on the setup model, and the positional direction of the dental implant was determined.

**Figure 7:**
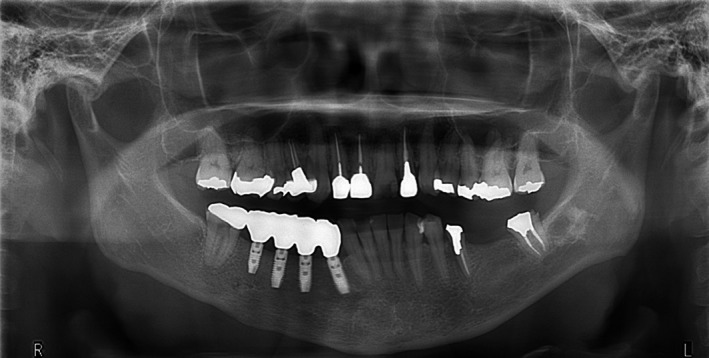
Orthopanoramic radiograph obtained 5 y after the operation

**Figure 8:**
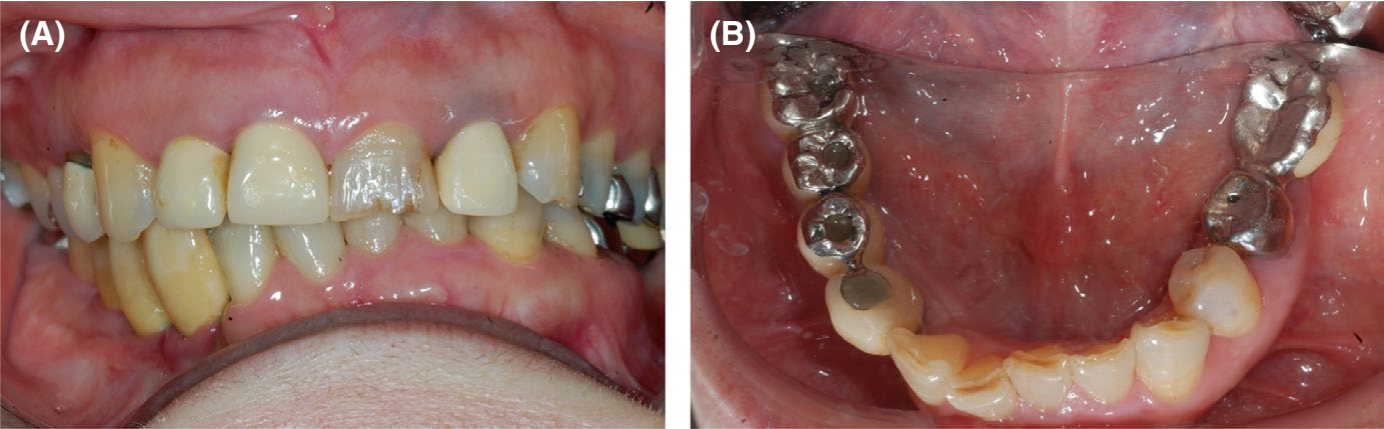
Intraoral photographs obtained 3 y after prosthesis installation. A, Frontal view. B, Occlusal view

In November 2014, with the patient under general anesthesia, the mini‐plate was removed from the grafted bone, and the dental implants were placed. We placed four dental implants using a surgical stent (for teeth 43, 44, and 45, 3.5 mm in diameter and 11 mm in length and for tooth 46, 3.5 mm in diameter and 9 mm in length). (Astra Tech Implant System, Dentsply Sirona Inc). All the implants had the appropriate insertion torque value. Five months after primary surgery, vestibuloplasty (vestibular extension with periosteal separation procedures) was performed under local anesthesia because oral hygiene management was difficult due to narrow oral vestibule and mobile mucosa. During the second procedure conducted in June 2015, we confirmed osseointegration in all four implants. The patient wore the final fixed prosthesis in October 2015. We regularly performed maintenance therapy to manage tumor recurrence (CT examination every 6‐12 months) and to check or provide care for oral hygiene every 3‐4 months (Figures 7 and 8). At the time of writing, the tumor had not recurred, and there was no sign of abnormal bone absorption or disintegration of the dental implants. The patient's oral hygiene is good, with no implant movement or irritation of the mucosa around the implant. She is satisfied with the results both aesthetically and functionally.

## 3 DISCUSSION

The radical treatment of ameloblastoma is surgical resection.^2^, ^3^ Segmental mandibulectomy causes lower face deformation and asymmetry, and deviation of the mandible causes dysfunctions in mastication, swallowing, and articulation. They greatly reduce the quality of life. Therefore, if a continuity defect exists in the mandible, some reconstruction is desirable and is essential for maintaining social life.^4^, ^5^ Numerous techniques have been reported for the reconstruction of mandibular defects.^6^, ^7^, ^8^, ^9^ The choice of the reconstruction technique to be used depends on whether the reconstruction is to be performed immediately or later, the condition of the recipient site, the amount of bone and soft tissue required, and the length of the defect. Currently, mandibular reconstruction commonly involves bone graft, and the bone graft selected can be completely detached from its original blood supply and be revascularized (examples of such types of bone are the fibula, scapula, and the iliac crest).^10^, ^11^, ^12^

The vascularized bone flaps can be transplanted as living bone cells because blood circulation resumes instantly through vascular anastomosis.^13^ Vascularized bone flaps can be adjusted to receive blood from the main vessel, and so it can be used for soft tissue. The disadvantage is that the donor site is highly invasive and requires a long operation time, so the elderly and patients with vascular fragility and poor general condition are not indicated. On the other hand, nonvascularized bone grafts have also been useful in the reconstruction of the mandible.^14^, ^15^, ^16^ Several donor sites can provide such bone grafts.^17^, ^18^ Nonvascularized bone grafts can be easily placed because they do not require the special microvascular surgery technique, unlike with vascularized bone flaps. Nonvascularized bone grafts have an advantage that the operation time and hospitalization period associated with them can be shortened compared with vascularized bone flaps.^19^ The iliac bone is often used in the restoration of the mandible.^4^, ^20^ The curvature of the iliac crest is similar to that of the mandible and can also be used in reconstruction involving the mandible angle. Nonvascularized iliac bone grafts yield good results if the lateral defect is <5 or 6 cm 21 and are suitable for the placement of osseointegrated implants.^22^

The main purpose of mandibular reconstruction after mandibulectomy is good mastication and restoration of swallowing function without limiting eating behavior. The first phase of well swallowing begins with the stability of the mandibular supported by pairing occlusion. Now, it is unquestionable that the gold standard of functional recovery of missing teeth with mandibular resection is a fixation prosthesis by osteointegrated implant.^23^ In order to achieve that outcome, the following consideration is necessary at the time of bony reconstruction,^24^ that is, contrasting mandibular shape, OI implantable thickness and height, the occlusal relationship, in a position where the dental implant can establish an ideal occlusal relationship. Preoperative planning with three‐dimensional digital images and an occlusal dental model are necessary to achieve this.

In reconstruction with an autogenous free bone graft, it is very difficult to create an ideal three‐dimensional alveolar bone structure for placement of the osseointegrated implant. Dumbach et al^25^ reported a reconstruction technique in which they used a titanium mesh tray and autologous iliac bone marrow (particulate cancellous bone and marrow [PCBM]). Preformed trays have been replaced by custom‐made trays, and some clinical usefulness has been reported.^26^, ^27^, ^28^ The advantage of PCBM transplantation over autogenous free bone grafts is that the postoperative injury at the donor site is mild and special surgical procedures such as microsurgery are not needed.^29^

For the placement of osseointegrated implant, the thickness of the bone must be 5 mm or more, and the height of the jaw bone should be preferably 10 mm or more. Several reports have suggested the possibility of using short implants, that is, survival rate comparable with long implants and efficacy compared with bone augmentation.^30^, ^31^ Currently, the long‐term efficacy of short implants is uncertain, and it is prudent to use implants that are as long as the root length.

There is no answer to the question: When should the dental implant be transplanted in the reconstructed bone? Vascularized bone grafts may allow simultaneous implantation of implants, as circulation resumes immediately. The nonvascularized grafts in the first phase are mostly necrotic because blood flow is blocked. The combination of HBO therapy promotes reorganization by capillary invasion during free bone grafting to stimulate pathways involved in angiogenesis.^32^ If HBO therapy is possible, it may be a contributor to treatment outcome and may shorten treatment duration. Because it aids in engraftment and provides good acceptance conditions for dental implants by suppressing the resorption of bone grafts. Shirota et al suggest about placement time of dental implant as follows: In the free iliac bone graft, it is desirable after successful grafting^33^ and in the case of reconstruction of segmental resection of the mandibular, the waiting period of 2 years or more after the operation is necessary.^34^ Freilich et al stated that healing of the grafts is a prerequisite and the period is 4‐8 months after surgery.^35^ Empirically, we know the size of the bone graft will shrink until 6 months after surgery and stabilize after 12 months. A similar incident was reported in a study on transplant bone size changes observed during X‐ray examination,^36^ which showed bone resorption in the early stage of transplantation (post 2 or 3 months) and bone formation image observed post 6 months. We believe that the appropriate time for implant placement is desirable when the appearance of cortical bone‐like bone formation around the graft bone is desirable, as it is advantageous for the initial fixation of the implant. An increase in cortical bone‐like calcification in the periphery of the bone graft in CT coronal sectional images is an important sign. On the other hand, in PCBM, bone marrow cells are transplanted as live cells and engraftment. Placement of dental implants on the PCBM site may be possible earlier than NVBG.

## CONFLICT OF INTEREST

The authors declare that they have no conflict of interest related to any product used in this study.

## AUTHOR CONTRIBUTIONS

Toshiyuki Kataoka, Toshihiro Okamoto, and Tomohiro Ando: conceived the ideas. Toshiyuki Kataoka: designed the study and wrote the initial draft of the manuscript. All other authors have contributed to data collection and interpretation and critically reviewed the manuscript. All authors have approved the final version of the manuscript and agree to be accountable for all aspects of the work in ensuring that questions related to the accuracy or integrity of any part of the work are appropriately investigated and resolved.
